# Associated factors of suicide attempt among patients with major depressive disorder in Vietnam

**DOI:** 10.1192/j.eurpsy.2025.2363

**Published:** 2025-08-26

**Authors:** S. Nguyen Thi Thu, T. Nguyen Dao Uyen

**Affiliations:** 1 Psychiatry, University of Medicine and Pharmacy at HCM City; 2 Neuropsyatry, Nguyen Tri Phuong Hospital; 3 Family Medicine, University of Medicine and Pharmacy at HCM City, Ho Chi Minh city, Viet Nam

## Abstract

**Introduction:**

Suicide attempt is a person’s suicidal behavior that does not result in death and may or may not result in injury. Understanding the factors associated with suicide attempts in patients with major depressive disorder is important to predicting future suicide attempts.

**Objectives:**

To identify the associated factors with suicide attempts in patients with MDD at the University Medicine Center in Vietnam.

**Methods:**

This cross-sectional analytical study was conducted in the psychiatry clinic of the University Medical Center of Ho Chi Minh City, Vietnam, from March to October 2023. Individuals aged 18 and more diagnosed with major depressive disorder as per DSM-5 TR were included. Exclusion criteria were current psychosis, severe intellectual disabilities, and acute medical illnesses.

Participants were interviewed using a questionnaire including sociodemographic criteria, clinical information, and the Hamilton Depression Rating Scale (HDRS).

**Results:**

We collected 151 participants. The average age of participants was 41.3±15.5 years, and they were predominantly female (78.8%) and living in urban areas (62.9%). Nearly four fifths (79.5%) of patients are currently in severe depression. The prevalence of suicide attempts in the lifetime and past 3-months were 7.9% and 5.3% respectively.

In univariate logistic regression analysis of sociodemographic factors and clinical features of depression associated with suicidal attempt among individuals with major depressive disorder, we found that young age (OR=0,91; p=0,004), single status (OR=0,09; p=0,002), early onset of illness (OR=0,91; 95% CI 0,85-0,97), and severe depression as measured by the total HDRS score (OR= 1,19; 95% CI 1,06-1,34). In particular, the risk of a suicide attempt was 72 times higher in patients with a history of self-harm (OR=72,22; 95% CI 13,71-380,49). There was no association between gender, area, education level, cohabitation status and employment status with lifetime prevalence of suicide attempts. After adjusting for covariates using a multivariable logistic regression model, only the severity of depressive episode and history of self-harm remained significantly associated with suicide attempts.

**Conclusions:**

Suicide attempts were significantly high among patients of major depressive disorder in Vietnam. The severity of depression and previous self-harm was significantly associated with it. There is a need for more research and a better understanding of the associated factor with suicide attempts in this population which in turn could lead to the development and implementation of effective preventive interventions.

**Disclosure of Interest:**

None Declared

